# BK K^+^ channel blockade inhibits radiation-induced migration/brain infiltration of glioblastoma cells

**DOI:** 10.18632/oncotarget.7423

**Published:** 2016-02-16

**Authors:** Lena Edalat, Benjamin Stegen, Lukas Klumpp, Erik Haehl, Karin Schilbach, Robert Lukowski, Matthias Kühnle, Günther Bernhardt, Armin Buschauer, Daniel Zips, Peter Ruth, Stephan M. Huber

**Affiliations:** ^1^ Department of Pharmacology, Toxicology and Clinical Pharmacy, University of Tübingen, Tübingen, Germany; ^2^ Department of Radiation Oncology, University of Tübingen, Tübingen, Germany; ^3^ Department of General Pediatrics, Oncology/Hematology, University of Tübingen, Tübingen, Germany; ^4^ Department of Pharmaceutical/Medicinal Chemistry II, University of Regensburg, Regensburg, Germany; ^5^ Dr. Margarete Fischer-Bosch-Institute of Clinical Pharmacology, University of Tübingen, Tübingen, Germany

**Keywords:** glioma, radiation therapy, patch-clamp recording, fura-2 Ca2^+^ imaging, transfilter migration

## Abstract

Infiltration of the brain by glioblastoma cells reportedly requires Ca^2+^ signals and BK K^+^ channels that program and drive glioblastoma cell migration, respectively. Ionizing radiation (IR) has been shown to induce expression of the chemokine SDF-1, to alter the Ca^2+^ signaling, and to stimulate cell migration of glioblastoma cells. Here, we quantified fractionated IR-induced migration/brain infiltration of human glioblastoma cells *in vitro* and in an orthotopic mouse model and analyzed the role of SDF-1/CXCR4 signaling and BK channels. To this end, the radiation-induced migratory phenotypes of human T98G and far-red fluorescent U-87MG-Katushka glioblastoma cells were characterized by mRNA and protein expression, fura-2 Ca^2+^ imaging, BK patch-clamp recording and transfilter migration assay. In addition, U-87MG-Katushka cells were grown to solid glioblastomas in the right hemispheres of immunocompromised mice, fractionated irradiated (6 MV photons) with 5 × 0 or 5 × 2 Gy, and SDF-1, CXCR4, and BK protein expression by the tumor as well as glioblastoma brain infiltration was analyzed in dependence on BK channel targeting by systemic paxilline application concomitant to IR. As a result, IR stimulated SDF-1 signaling and induced migration of glioblastoma cells *in vitro* and *in vivo*. Importantly, paxilline blocked IR-induced migration *in vivo*. Collectively, our data demonstrate that fractionated IR of glioblastoma stimulates and BK K^+^ channel targeting mitigates migration and brain infiltration of glioblastoma cells *in vivo*. This suggests that BK channel targeting might represent a novel approach to overcome radiation-induced spreading of malignant brain tumors during radiotherapy.

## INTRODUCTION

Glioblastoma multiforme consists of cells with a highly migratory phenotype that may “travel” long distances throughout the brain [1]. Primary foci of glioblastoma usually show a characteristic diffuse and net-like brain infiltration which represents a major challenge for surgical tumor resection as well as for adequate coverage by radiotherapy [2]. This migratory phenotype in concert with a pronounced resistance to radiotherapy and chemotherapy probably contributes to frequent therapy failure and bad prognosis observed in the vast majority of patients with glioblastoma.

Sublethal IR has been demonstrated *in vitro* and/or in rodent tumor models to induce migration, metastasis, invasion and spreading of a variety of tumor entitites. In particular, a plethora of *in vitro* and *in vivo* studies suggest that IR induces migration of glioblastoma cells (for review see [3, 4]). Three-dimensional-glioblastoma *in vitro* models, however, could not confirm this phenomenon [5] and whether or not IR induces migration of glioblastoma cells *in vivo* is still under debate.

If IR-induced migration, however, reaches relevant levels during fractionated radiotherapy of glioblastoma patients it might boost glioblastoma brain infiltration and - in the worst case - evasion of glioblastoma cells from the target volume of the radiotherapy. Along those lines, the chemokine SDF-1 (stromal cell-derived factor-1, CXCL12) via its receptor CXCR4 [6–8] stimulates migration of glioblastoma cells [9]. IR reportedly induces the expression of SDF-1 in different tumor entities including glioblastoma [10–13] as well as in normal brain tissue [7].

Collectively, these findings suggest that IR-induced migration may contribute to therapy resistance of glioblastoma. The present study, therefore, aimed to provide a quantitative analysis of IR-induced migration/brain infiltration in an orthotopic *xeno*graft model of human glioblastoma. Importantly, a previous *in vitro* study of our group disclosed IR-induced BK K^+^ channel activation as a key event in IR-induced migration. Since BK channel blockade by paxilline, a toxin of the fungus *Penicillium paxilli*, suppresses IR-induced migration *in vitro* [14] the present study further tested whether glioma BK channel targeting with paxilline might be a powerful strategy to suppress IR-induced migration of glioblastoma cells *in vivo*. Our data strongly suggest that fractionated IR stimulates glioblastoma migration/infiltration *in vivo* via auto-/paracrine SDF-1 signaling and subsequent BK channel activation.

## RESULTS

Studies using human U-87MG glioblastoma cells to generate orthotopic mouse models report encapsulated and low brain infiltrative tumor growth [15]. Therefore, U-87MG glioblastoma seemed excellently suited for quantitative analysis of number and migration distances of individual glioblastoma cells. We used the U-87MG-Katushka clone stably transfected with the far-red fluorescent protein Katushka for histological glioblastoma cell tracking. The Katushka protein-expressing U-87MG cells were comparable to the wild type cells regarding growth kinetics and chemosensitivity against standard cytostatic drugs as shown in Supplementary Figure S1A–S1C. The BK inhibitor paxilline had no significant antiproliferative activity on U-87MG-Katushka cells upon long-term exposure at concentrations of up to 10 μM (Supplementary Figure S1D).

First, we studied *in vitro* both BK channel expression in U-87MG-Katushka cells and putative radiosensitizing effects of the BK channel inhibitor paxilline. Issuing the latter was plausible since pharmacological blockade of the BK-related Ca^2+^-activated IK channels reportedly radiosensitizes T98G and U-87MG glioblastoma cells [16]. Similar radiosensitizing action of paxilline would complicate the interpretation of any paxilline *in vivo* effect on tumor cell migration and brain infiltration.

**Figure 1:**
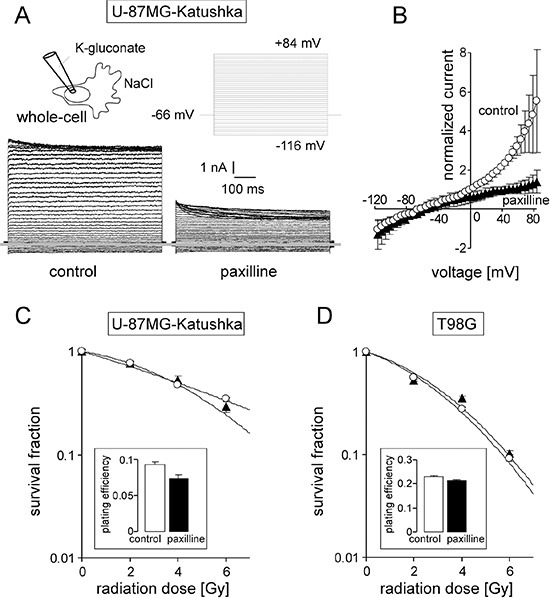
The glioblastoma cell lines T98G and U-87MG-Katushka functionally express BK Ca^2+^-activated K^+^ channels which, in contrast to IK channels, do not modulate radioresistance (**A**) Whole-cell current tracings recorded from the cell rear of a migrating U-87MG-Katushka cell before (left) und during (right) bath application of the BK channel inhibitor paxilline. Records were obtained in voltage-clamp mode with K-gluconate pipette- and NaCl bath solution. The applied pulse protocol is shown in the upper right, the grey line indicates zero current level. (**B**) Mean (± SE, *n* = 3) whole-cell current densities of migrating U-87MG-Katushka cells recorded as in (A) before (circles) and during paxilline administration (triangles). (**C, D**) Mean survival (± SE, *n =* 12–36) fraction of irradiated (0–6 Gy) U-87MG-Katushka (C) and T98G cells (D) as determined by delayed plating colony formation assay. Cells were irradiated and post-incubated (24 h) in the absence (open bars) or presence (closed triangles) of paxilline. The inserts show the plating efficiencies of both cell lines in the absence (open bars) or presence of paxilline (closed bars).

As described for T98G and the parental U-87MG cells [14], the U-87MG-Katushka clone functionally expressed BK channels. This was evident from whole-cell patch-clamp recordings with K-gluconate in the pipette and NaCl in the bath. U-87MG-Katuska cells exhibited large outward currents in the range of several nano-amperes (Figure 1A, left). These currents were outwardly rectifying and blocked by the BK channel inhibitor paxilline (Figure 1A right and 1B) indicative of functional expression of BK channels. To test for a radiosensitizing action of BK channel targeting, the influence of paxilline on clonogenic survival of irradiated U-87MG-Katushka and T98G cells was determined by delayed plating colony formation assays. In contrast to IK channel targeting [16], BK channel blockade by paxilline did not radiosensitize either glioblastoma cell models (Figure 1C and 1D).

**Figure 2:**
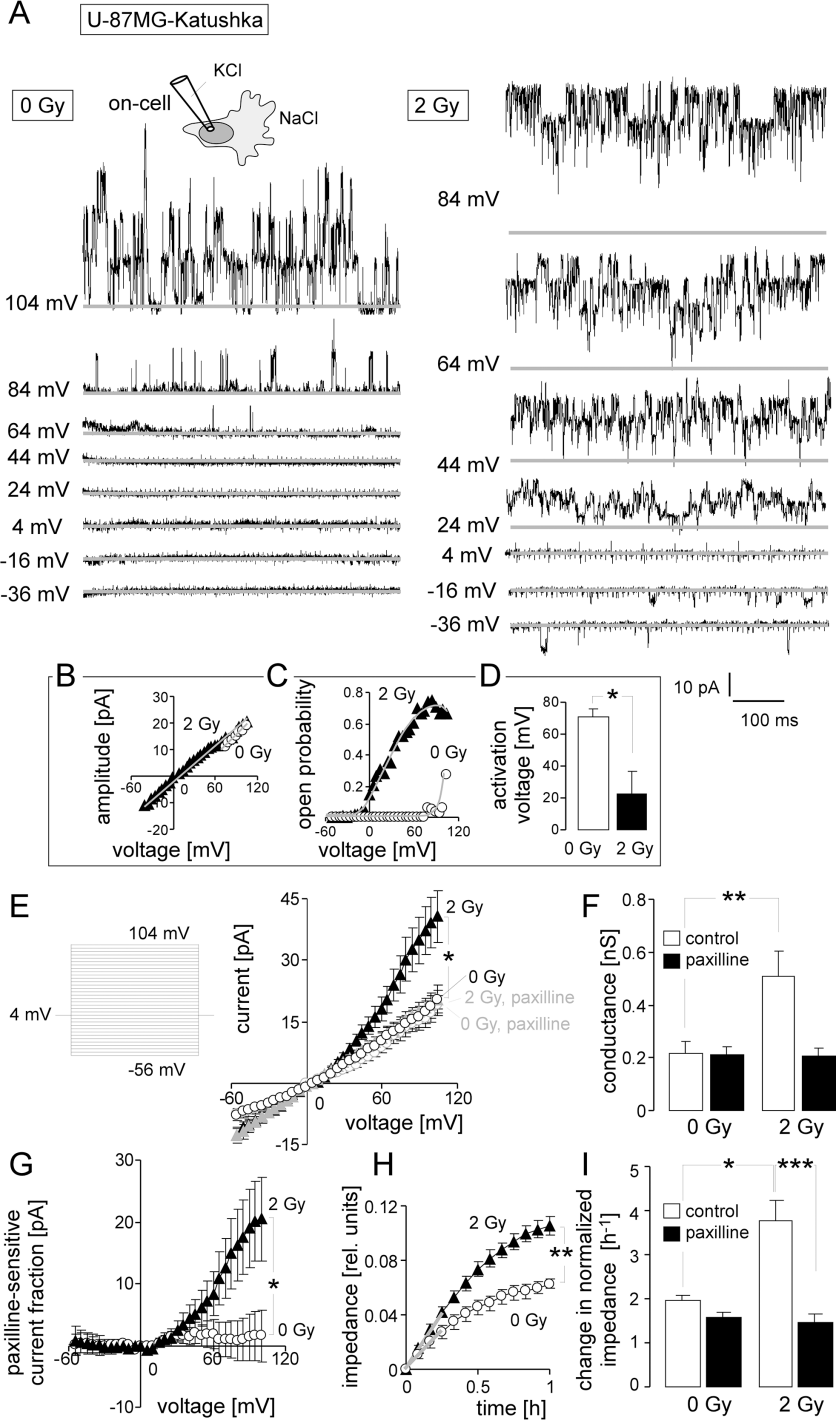
Ionizing radiation (IR) stimulates BK K^+^ channel-dependent migration of U-87MG-Katushka cells (**A**) Single channel current transitions recorded in on-cell mode at different holding potentials (as indicated) with KCl pipette- and NaCl bath solutions from a control (left) and an irradiated (3 h after 2 Gy) U-87MG-Katushka cell. The voltage-dependent increase in open probability is shifted towards more negative potentials in the irradiated as compared to the control cell. (**B, C**) Dependence of the mean unitary current transition (B) and open probability (P_o_, C) on the voltage of the channels recorded in the control (open circles) and the irradiated cell (closed triangle) shown in (A). The channels exhibiting a unitary conductance of g ≈ 200 pS and a depolarization-induced increase of P_o_ typically for BK channels. (**D**) Mean (± SE, *n =* 9) minimal depolarizing voltage evoking BK channel activity in 0 Gy- (open bar) or 2 Gy-irradiated (closed bar) U-87MG-Katushka cells. (**E**) Mean (± SE, *n =* 8–15) macroscopic on-cell currents recorded as in (A) from control (open circles) and irradiated (2 Gy, closed triangles) U-87MG-Katushka cells. Records were obtained in the absence (black symbols) or presence (grey symbols) of the BK inhibitor paxilline. (**F**) Mean (± SE) conductance of the clamped membrane patch as calculated from (E) for the outward currents for control and irradiated cells in the absence (open bars) or presence (closed bars) of paxilline. (**G**) Mean (± SE) paxilline-sensitive current fractions of control (open circles) and irradiated cells (closed triangles, data from E). (**H**) Mean (± SE, *n =* 4) impedance as measure of transfilter migration of cells irradiated with 0 Gy (open circles) or 2 Gy (closed triangles). The experiment started at 2 h after IR. (**I**) Mean (± SE, *n =* 9–24) normalized migration velocity as calculated for the first 0.25 h of transfilter migration (slopes in H shown by the grey lines) in control (0 Gy) and irradiated (2 Gy) cells recorded in the absence (open bars) or presence of the BK inhibitor paxilline (closed bars). *, ** and *** indicate *p* ≤ 0.05, *p* ≤ 0.01, and *p* ≤ 0.001, respectively, ANOVA (E, F, I) or two-tailed Welch corrected *t*-test (D, H).

Reportedly, IR stimulates *in vivo* the expression of the chemokine SDF-1 by the glioma invasion front [13]. Therefore, U-87MG-Katushka and T98G were tested *in vitro* for IR-induced BK channel activity, transfilter migration and the role of SDF-1 signaling herein in order to define radiation-induced signaling events upstream of BK channel activation. In on-cell patch-clamp recordings (KCl pipette- and NaCl bath solution) from U-87MG-Katushka cells (Figure 2A), large conductance ion channels (unitary conductance, g ≈ 200 pS, Figure 2B) became increasingly active with increasing positive voltage. In irradiated cells (2 Gy, 2–4.5 h after IR), channel activity was observed at highly significantly lower clamp-voltage than in non-irradiated cells (Figure 2A, right, Figure 2C, closed triangles and Figure 2D, closed bar). In on-cell mode, a clamp-voltage between pipette and bath solution of 0 mV is recording the transmembrane currents at physiological membrane potential. Therefore, the current transitions at 0 mV observed in irradiated cells indicated the activity of the large conductance ion channel at physiological membrane potential. In unirradiated control cells, in contrast, channel activity was triggered only by clamp-voltages above +50 mV (Figure 2D, open bar). Accordingly, mean macroscopic on-cell outward currents in irradiated cells exceeded significantly that of control cells by twofold (Figure 2E, black symbols and Figure 2F, open bars). Notably, paxilline (5 μM; Figure 2E, grey symbols and Figure 2F, closed bars) blocked about half of the outward current in irradiated cells while having no effect in control cells. The paxilline-sensitive current fractions of irradiated cells showed typical outward rectification (Figure 2G, closed triangles). In combination, voltage-dependence of open probability, high unitary conductance and paxilline-sensitivity defined the large conductance channel as BK K^+^ channel. Importantly, BK channels were active in irradiated cells at physiological membrane potential suggesting their functional significance for the irradiated glioblastoma cells.

BK channel activation in irradiated U-87MG-Katushka cells was paralleled by significantly faster chemotaxis when compared to unirradiated control cells as determined by FCS gradient-stimulated transfilter migration assays (Figure 2H and 2I, open bars). The BK channel inhibitor paxilline (5 μM) did not affect the basal fraction of migrating cells, whereas the IR-induced migration was completely abolished (Figure 2I, closed bars). Together, these data indicate radiation-induced migration in U-87MG-Katushka cells depending on IR-induced BK channel activity.

**Figure 3:**
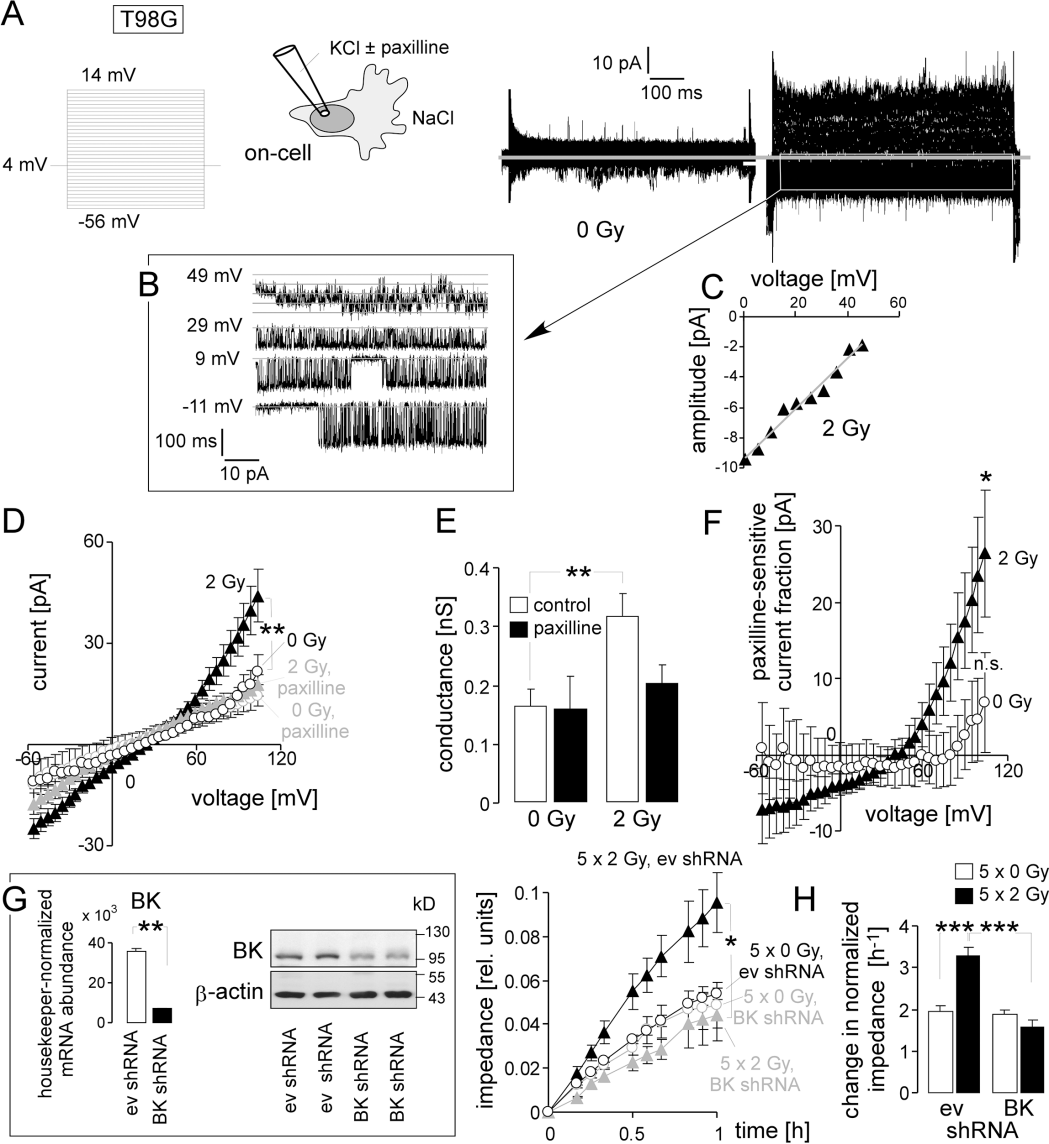
IR stimulates BK channel activity and BK channel-dependent migration of human T98G glioblastoma cells (**A**) Macroscopic on-cell currents recorded at different voltages (as indicated) with KCl pipette- and NaCl bath solutions from a control (left) and an irradiated T98G cell. (**B, C**) Single channel current transitions at different holding potentials (B) and dependence of the current amplitude on voltage (C) extracted from the current tracings in (A, right) indicate a voltage-dependent activation and a unitary conductance characteristic for BK channels. (**D**) Mean (± SE, *n =* 8–34) macroscopic on-cell currents recorded as in (A) from control (open circles) and irradiated (2 Gy, closed triangles) T98G cells. Records were obtained in the absence (black) or presence (grey) of the BK inhibitor paxilline. (**E**) Mean (± SE) conductance of the clamped membrane patch as calculated from (D) for the outward currents for control and irradiated cells in the absence (open bars) or presence (closed bars) of paxilline. (**F**) Mean (± SE) paxilline-sensitive current fractions of control (open circles) and irradiated (closed triangles) cells (data from D). (**G**) Knockdown of BK channels in T98G cells abrogates radiation-induced migration. Mean (± SE, *n* = 4) impedance as measure of transfilter migration of empty vector control (ev, black) or BK-specific shRNA-expressing T98G cells (grey) irradiated with 5 × 0 Gy (open circles) or 5 × 2 Gy (closed triangles). The experiment started 24 h after the last IR fraction. The insert (left) shows the housekeeper-normalized BK-encoding mRNA abundance (left) as well as BK and β-actin protein abundances of T98G cells stably transduced with empty vector control (ev) or BK-specific shRNA. (**H**) Mean (± SE, *n* = 8) normalized migration velocity as calculated from the data in (G) for the first 0.25 h of transfilter migration in control (0 Gy, open bars) and irradiated (2 Gy, closed bars) cells expressing non or BK-specific shRNA. *, ** and *** indicate *p* ≤ 0.05, *p* ≤ 0.01, and *p* ≤ 0.001, respectively, ANOVA (D, G, H) or Welch corrected *t*-test (G). * and n. s. in Figure F indicate significantly (*p* ≤ 0.05) and not significantly different from 0, respectively, two-tailed one-sample *t*-test.

To confirm previously published data on paxilline-sensitive IR-induced migration [14], irradiated (0 or 2 Gy, 2–4.5 h after IR) T98G cells were on-cell patch-clamp recorded with KCl in the pipette and NaCl bath solution. Similar to the U-87MG-Katushka model, irradiated T98G glioblastoma cells showed voltage-dependent activity of large conductance (g ≈ 200 pS) channels (Figure 3A–3C). Channels were active in irradiated T98G cells at physiological membrane potential (i.e., 0 mV clamp-voltage, Figure 3B) and generated an outwardly rectifying macroscopic on-cell current (Figure 3D, black closed triangles, and Figure 3E, 3rd bar) that was significantly larger than that of the unirradiated T98G control cells (Figure 3D, black open circles, and Figure 3E, 1st bar). The BK inhibitor paxilline blocked the IR-induced outward current (Figure 3D, grey closed triangles and Figure 3E, 4th bar) and the paxilline-sensitive current fraction showed typical outward rectification (Figure 3F, closed triangles) indicative of IR-induced BK channel activation. Unirradiated T98G control cells, in contrast, did not exhibit significant paxilline-sensitive outward currents even at high clamp voltages (Figure 3D, open circles, Figure 3E, 1st and 2nd bar, and Figure 3F, open circles).

IR-induced migration (Figure 3G, black symbols and Figure 3H, 1st and 2nd bar) required BK activation, because shRNA-mediated knockdown of BK (Figure 3G, insert) significantly decreased migration velocity of irradiated cells to the control values while not affecting basal migration of unirradiated control cells (Figure 3H, grey symbols and Figure 3H, 3rd and 4th bar). These results suggest that the previously reported IR-induced and paxilline-sensitive migration of T98G cells [14] is mediated by IR-induced BK channel activation.

**Figure 4:**
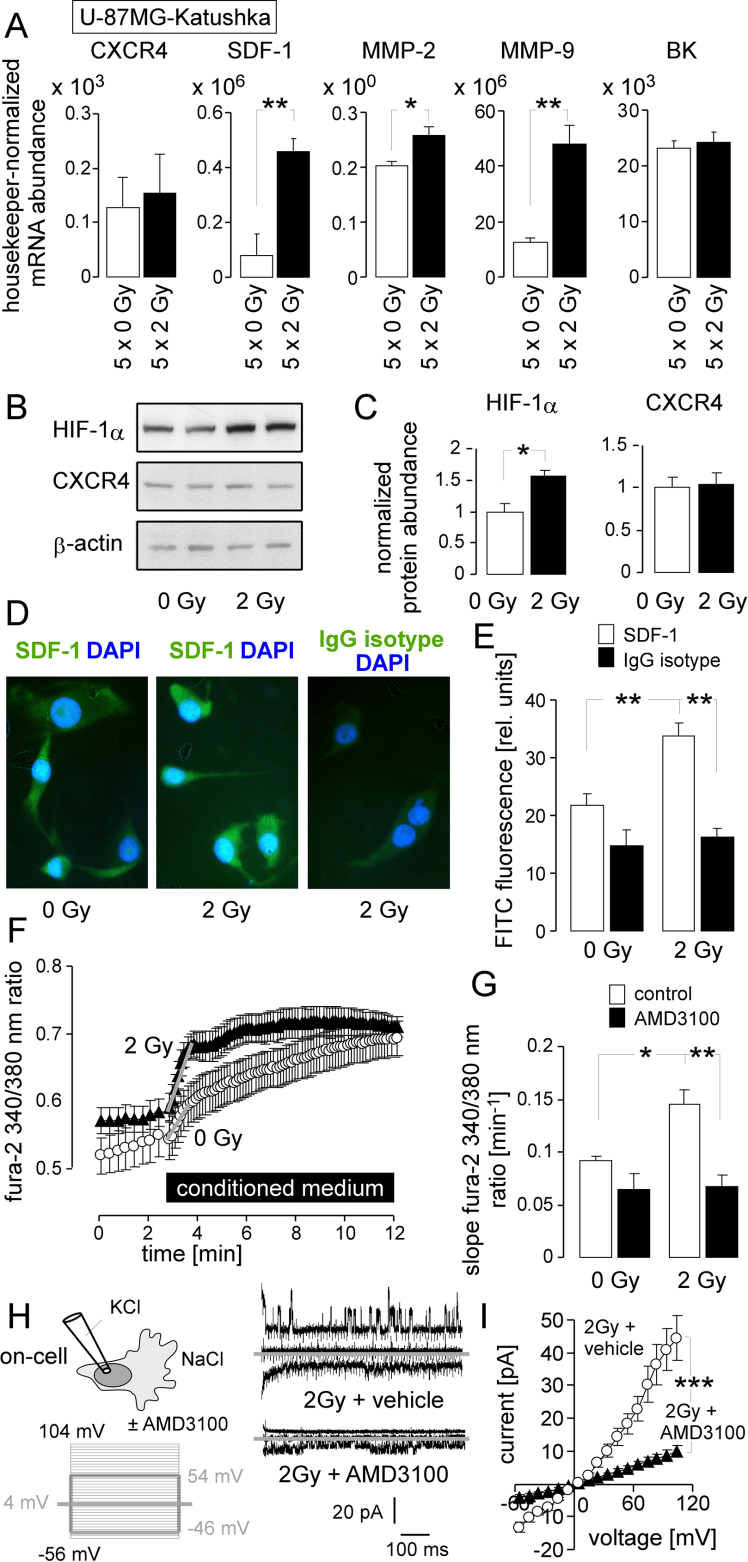
IR stimulates a migratory and invasive phenotype in U-87MG-Katushka cells probably via stabilization of HIF-1α, upregulation of SDF-1, CXCR4-mediated Ca^2+^ signaling and BK channel activation (**A**) Mean (± SE, *n* = 5) mRNA abundances of U-87MG-Katushka cells fractionated irradiated with 5 × 0 Gy (open bars) or 5 × 2 Gy (closed bar, mRNA was extracted 24 h after the last IR fraction). Shown are the mRNAs encoding for the chemokine receptor CXCR4, the chemokine SDF-1 (CXCL12), the matrix metalloproteinase MMP-2 and MMP-9, as well as for the BK channel. (**B**) Immunoblots of two lysates each prepared from U-87MG-Katuskha cells irradiated with a single dose of 0 Gy (left) or 2 Gy (2 h after IR, right) probed against HIF-1α, CXCR4 and the loading control β-actin. (**C**) Mean (± SE, *n* = 4) β-actin-normalized HIF-1α (left) and CXCR4 (right) protein abundance in 0 Gy (open bars) or 2 Gy (2 h after IR, closed bars) U-87MG-Katushka cells. (**D**) Immunofluorescence micrographs of 0 Gy- (left) or 2 Gy (2 h after IR, middle and right) stained with an anti-SDF-1 (left and middle) or the IgG isotype control antibody (right) as detected with a FITC-coupled secondary antibody (green) and co-stained with the DNA-specific dye DAPI (blue). (**E**) Mean (± SE) FITC fluorescence intensity of anti-SDF-1 (open bars; *n* = 286–364) or IgG isotype antibody-stained cells from 0 Gy (left, *n* = 42–75) or 2 Gy irradiated U-87MG-Katushka cells. (**F**) Mean (± SE, *n* = 36–60) fura-2 340/380 nm fluorescence ratio as measure of cytosolic free Ca^2+^ concentration (_free_ [Ca^2+^]_i_) recorded in U-87MG-Katushka cells before and during superfusion with conditioned medium harvested from U-87MG-Katushka cultures 2 h after IR with 0 Gy (open circles) or 2 Gy (closed triangles). (**G**) Mean (± SE, *n* = 24–60) increase in _free_ [Ca^2+^]_i_ as determined by the slope (grey lines in F) of the conditioned medium-evoked rise in the 340/380 nm ratio. The conditioned medium harvested from 0 Gy (left) or 2 Gy (right) irradiated U-87MG-Katushka cells was administered without (open bars) or together with the CXCR4 antagonist AMD3100 (closed bars). (**H**) AMD3100 prevents IR-induced induction of BK channel activity in U-87MG-Katushka cells. On-cell current tracings of irradiated cells (2 Gy, 2 h after IR) irradiated and post-incubated in the absence (top) or presence of AMD3100. Macroscopic on-cell currents were obtained with KCl pipette- and NaCl bath solutions in the absence of AMD3100 as described in Figure 2. Only currents evoked by voltage sweeps to −46, 4, and 54 mV are shown. (**I**) Dependence of mean (± SE, *n* = 16) macroscopic on-cell currents on voltage recorded as in (H) from vehicle- (open circles) or AMD3100-pretreated (closed triangles) irradiated U-87MG-Katushka cells. *, ** and *** indicate *p* ≤ 0.05, *p* ≤ 0.01, and *p* ≤ 0.001, respectively, two-tailed (Welch)-corrected *t*-test in (A, C, I) and ANOVA in (G, E).

To estimate, whether IR-induced migration might be associated with an hyperinvasive phenotype and to identify radiation-triggered signaling events, abundances of selected mRNAs were compared between fractionated irradiated (5 × 2 Gy) and control (5 × 0 Gy) U-87MG-Katushka cells. Specifically, mRNAs encoding for BK, the matrix metalloproteinases MMP-2 and MMP-9, the chemokine SDF-1 (CXCL12) and the SDF-1 receptor CXCR4 were analyzed. As shown in Figure 4A, fractionated IR did not alter BK or CXCR4 mRNA but significantly increased the abundance of MMP-2, MMP-9, and SDF-1 mRNA collectively pointing towards an IR-induced hyperinvasive phenotype.

The transcription factor hypoxia-inducible factor-1α (HIF-1α) has been reported to up-regulate CXCR4 and SDF-1 expression (for review see [6]). In U-87MG-Katushka, IR (1 × 2 Gy, 2 h after IR) stabilized HIF-1α protein as shown by immunoblotting (Figure 4B, upper blot and Figure 4C, left). In accordance with the mRNA data (Figure 4A), HIF-1α stabilization was not associated with an IR-induced increase in CXCR4 chemokine receptor protein abundance (immunoblot in Figure 4B, middle and Figure 4C, right) but was paralleled by a significantly elevated SDF-1 immunofluorescence (Figure 4D, 4E).

To estimate the functional significance of SDF-1 signaling in IR-induced BK activation and migration, the effect of conditioned medium harvested from control and irradiated U-87MG-Katushka cells (2 h after IR) on Ca^2+^ signaling was determined in the presence and absence of the CXCR4 antagonist AMD3100 (1 μM). In fura-2 Ca^2+^ imaging experiments, conditioned medium from irradiated cells evoked a significantly faster rise in cytosolic _free_ [Ca^2+^]_i_ than conditioned medium from unirradiated control cells (Figure 4F and 4G, open bars). Importantly, AMD3100, when washed-in together with the conditioned medium, completely abolished the IR effect on _free_[Ca^2+^]_i_ (Figure 4G, closed bars). This suggests that IR induces enrichment of factors stimulating the CXCR4 signaling in the medium. Likewise, AMD3100 significantly blocked the BK channel activation by IR when applied before on-cell patch-clamp recording (Figure 4H, 4I). Together, these data suggest the involvement of SDF-1/CXCR4 signaling in IR-induced BK channel activation of U-87MG-Katushka cells.

**Figure 5:**
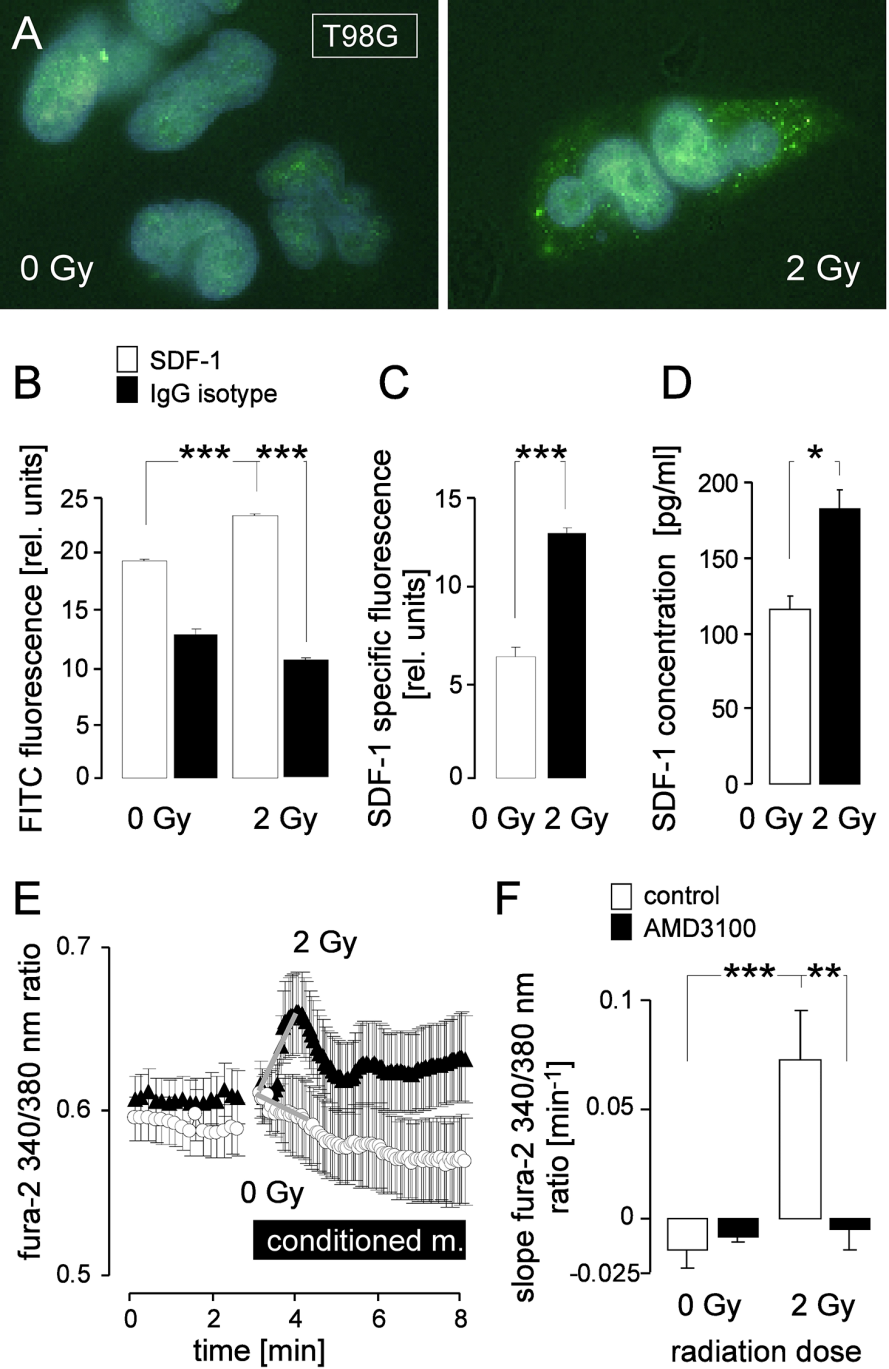
IR induces SDF-1 signaling of T98G cells (**A**) Immunofluorescence micrographs of 0 Gy (left) or 2 Gy (2 h after IR, right) irradiated cells stained with an anti-SDF-1 antibody and a FITC-coupled secondary antibody (green). (**B**) Mean (± SE) FITC fluorescence intensity of anti-SDF-1 (open bars, *n* = 286–364) or IgG isotype antibody-stained (closed bars, *n* = 42–75) cells and (**C**) SDF-1-specific fluorescence from 0 Gy (open bar) or 2 Gy irradiated T98G cells (closed bar). (**D**) Mean (± SE, *n* = 4) SDF-1 concentration in the medium of T98G cells 2 h after irradiaton with 0 Gy (open bar) or 2 Gy (closed bar). (**E-F**) CXCR4 chemokine receptor antagonist AMD3100 prevents IR-induced Ca^2+^ signaling. (E) Mean (± SE, *n* = 7–27) fura-2 340/380 nm fluorescence ratio as measure of cytosolic _free_ [Ca^2+^]_i_ recorded in T98G cells before and during superfusion with conditioned medium harvested from T98G cultures 2 h after IR with 0 Gy (open circles) or 2 Gy (closed triangles). (F) Mean (± SE) increase in _free_ [Ca^2+^]_i_ as determined by the slope (grey lines in E) of the conditioned medium-evoked rise in the 340/380 nm ratio. The conditioned media were administered without (open bars) or together with the CXCR4 antagonist AMD3100 (closed bars). *, **, and *** indicate *p* ≤ 0.05, *p* ≤ 0.01, and *p* ≤ 0.001, respectively, ANOVA in (B) and (F) and Welch-corrected *t*-test in C and (D).

Similarly to U-87MG-Katushka cells, T98G cells expressed CXCR4 and SDF1 mRNA and protein (RT-PCR and immunoblot data not shown). IR (2 Gy, 2 h after IR) induced a significant increase in SDF-1 protein abundance in T98G cells as determined by immunofluorescence microscopy (Figure 5A–5C). Conditioned medium from irradiated (2 Gy, 2 h after IR) T98G cells exhibited significantly higher SDF-1 concentrations than control cells as determined by ELISA (Figure 5D). Accordingly, superfusion of conditioned medium from irradiated T98G cells (2 Gy, 2 h after IR) stimulated a significant increase in _free_ [Ca^2+^]_i_ in T98G cells while medium from control cells did not (Figure 5E and 5F, open bars). The CXCR4 antagonist AMD3100 (1 μM) prevented the Ca^2+^ signals in T98G cells elicited by conditioned medium from irradiated cells (Figure 5F, closed bars). Together, these data suggest IR-induced SDF-1 signaling also in T98G cells.

**Figure 6:**
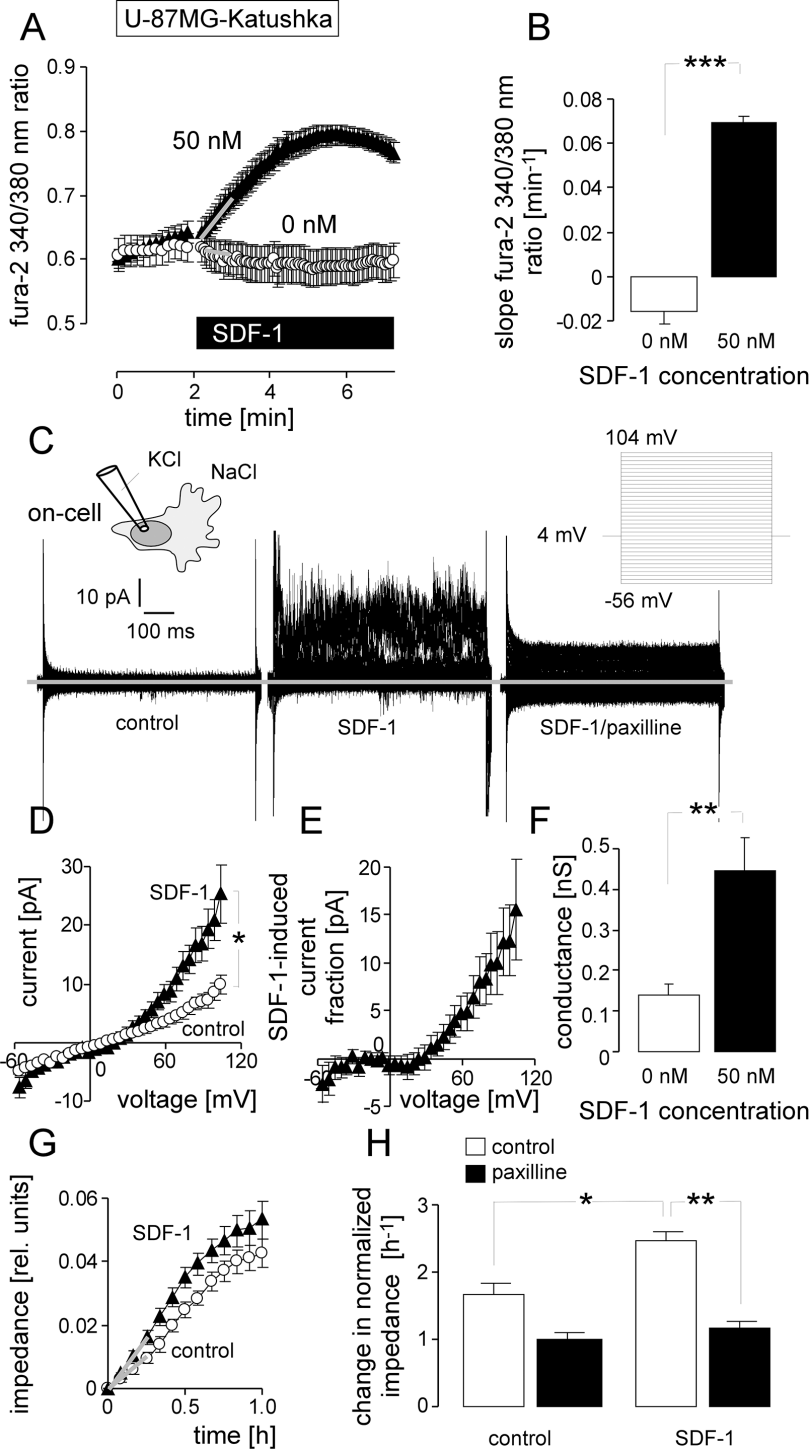
Stimulation with the chemokine SDF-1 mimics the effect of IR on BK channel activity and transfilter migration in U-87MG-Katushka cells (**A**) Mean (± SE, *n* = 19–23) fura-2 340/380 nm fluorescence ratio before and during superfusion with SDF-1 or control solution. (**B**) Mean (± SE) change in _free_ [Ca^2+^]_i_ as determined by the slope (grey lines in A) of the SDF-1- or control solution-evoked change in the 340/380 nm ratio. (**C**) On-cell current tracings recorded with KCl pipette- and NaCl bath solution from a U-87MG-Katuska cell before (left) and during bath application of SDF-1 (middle) and the BK inhibitor paxilline (right). (**D**) Mean (± SE, *n* = 10) macroscopic on-cell currents recorded as in (C) before (open circles) and during administration of SDF-1 (closed triangles). (**E**) Mean (± SE) SDF-1-stimulated current fraction (data from C). (**F**) Mean (± SE) conductance of the clamped membrane patch as calculated from (C) for the outward currents recorded in the absence (open bars) and presence of SDF-1 stimulation (closed bars). (**G**) Mean (± SE, *n* = 4) impedance as measure of transfilter migration of control (open circles) and SDF-1-stimulated (closed triangles) U-87MG-Katushka cells. (**H**) Mean (± SE, *n* = 9–24) normalized migration velocity in control and SDF-1-stimulated U-87MG-Katushka cells recorded in the absence (open bars) or presence (closed bars) of the BK inhibitor paxilline. *, ** and *** indicate *p* ≤ 0.05, *p* ≤ 0.01, and *p* ≤ 0.001, respectively, two-tailed Welch-corrected *t*-test in (B, D, F), ANOVA in (H).

To test, whether SDF-1 can mimic IR-induced BK channel activation and migration in U-87MG-Katushka cells, _free_ [Ca^2+^]_i_ was recorded during wash-in of SDF-1 (Figure 6A, 6B). Acute application of SDF-1 (50 nM) stimulated a significant long-lasting increase in _free_ [Ca^2+^]_i_. In addition, acute application of SDF-1 induced significant paxilline-sensitive outward currents in on-cell patch-clamp recordings (Figure 6C–6F). Remarkably, the SDF-1-stimulated current fraction was outwardly rectifying (Figure 6E) and closely resembled the radiation-induced currents (compare Figure 6E with Figure 2G, closed triangles) in voltage-dependence and absolute values. Finally, SDF-1 (50 nM) significantly increased transfilter migration of U-87MG-Katushka cells (Figure 6G and 6H, open bars). The BK channel inhibitor paxilline (5 μM) blocked the SDF-1 induced augmentation of transfilter migration without significantly inhibiting basal migration (Figure 6H, closed bars). In summary, SDF-1 very similarly to radiation stimulates migration that depends on BK channel activation.

**Figure 7:**
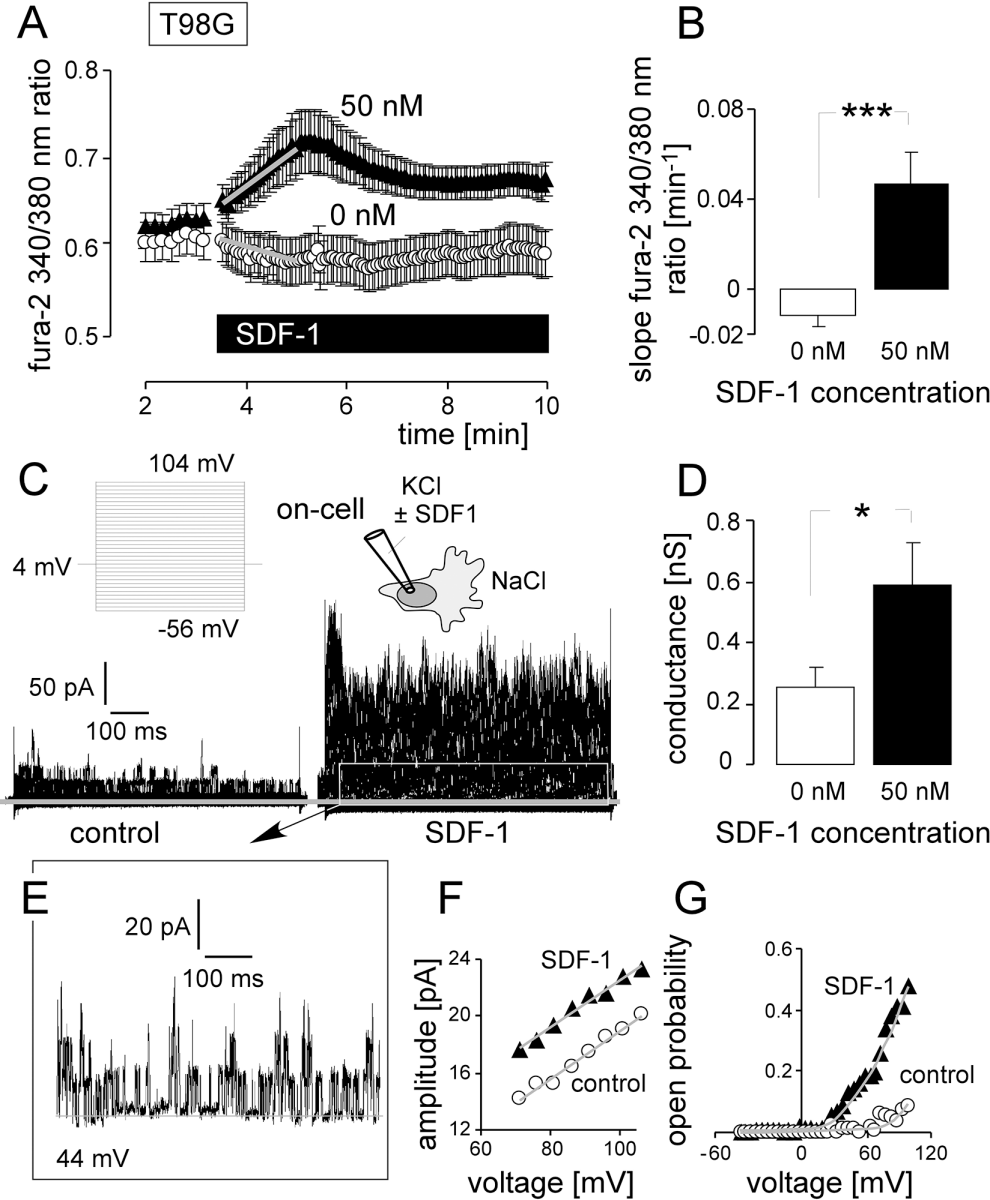
SDF-1 elicits Ca^2+^ signals and mimics the effect of IR on BK channel activity and migration in T98G cells (**A**) Mean (± SE, *n* = 21–36) fura-2 340/380 nm fluorescence ratio before and during superfusion with SDF-1 or control solution. (**B**) Mean (± SE) change in _free_ [Ca^2+^]_i_ as determined by the slope (grey lines in A) of the SDF-1- or control solution-evoked change in the 340/380 nm ratio. (**C**) On-cell current tracings recorded with KCl pipette- and NaCl bath solution from a control (left) and SDF-1-stimulated T98G cell (right). (**D**) Mean (± SE; *n* = 11) conductance of the clamped membrane patch as calculated from (C) for the outward currents. (**E-G**) Single channel current transitions (E) and dependence of the current amplitude on voltage (F) and open probability (G) of the control (open circles) and SDF-1-stimulated current tracings (closed triangles) shown in (C). * and *** indicate *p* ≤ 0.05 and 0.001, Welch-corrected *t*-test, respectively.

Analogous to U-87MG-Katushka, acute application of SDF-1 (50 nM) induced in T98G cells a long-lasting increase in _free_ [Ca^2+^]_i_, (Figure 7A, 7B) and an activation of macroscopic outward current in on-cell patch-clamp recordings (Figure 7C, 7D). Single channel analysis (Figure 7E) revealed BK-like large conductance channels (p ≈ 170 pS; Figure 7F) that activated increasingly with increasing voltage (Figure 7G). In summary, the *in vitro* data on the U-87MG-Katushka clone demonstrated IR-induced BK K^+^ channel-dependent migration similar to that observed in the present study and/or reported previously for T98G and the parental U-87MG cells [14]. Moreover, these *in vitro* experiments strongly suggest that IR-induced SDF-1 signaling is triggering upstream of BK at least part of the IR-induced migration.

**Figure 8:**
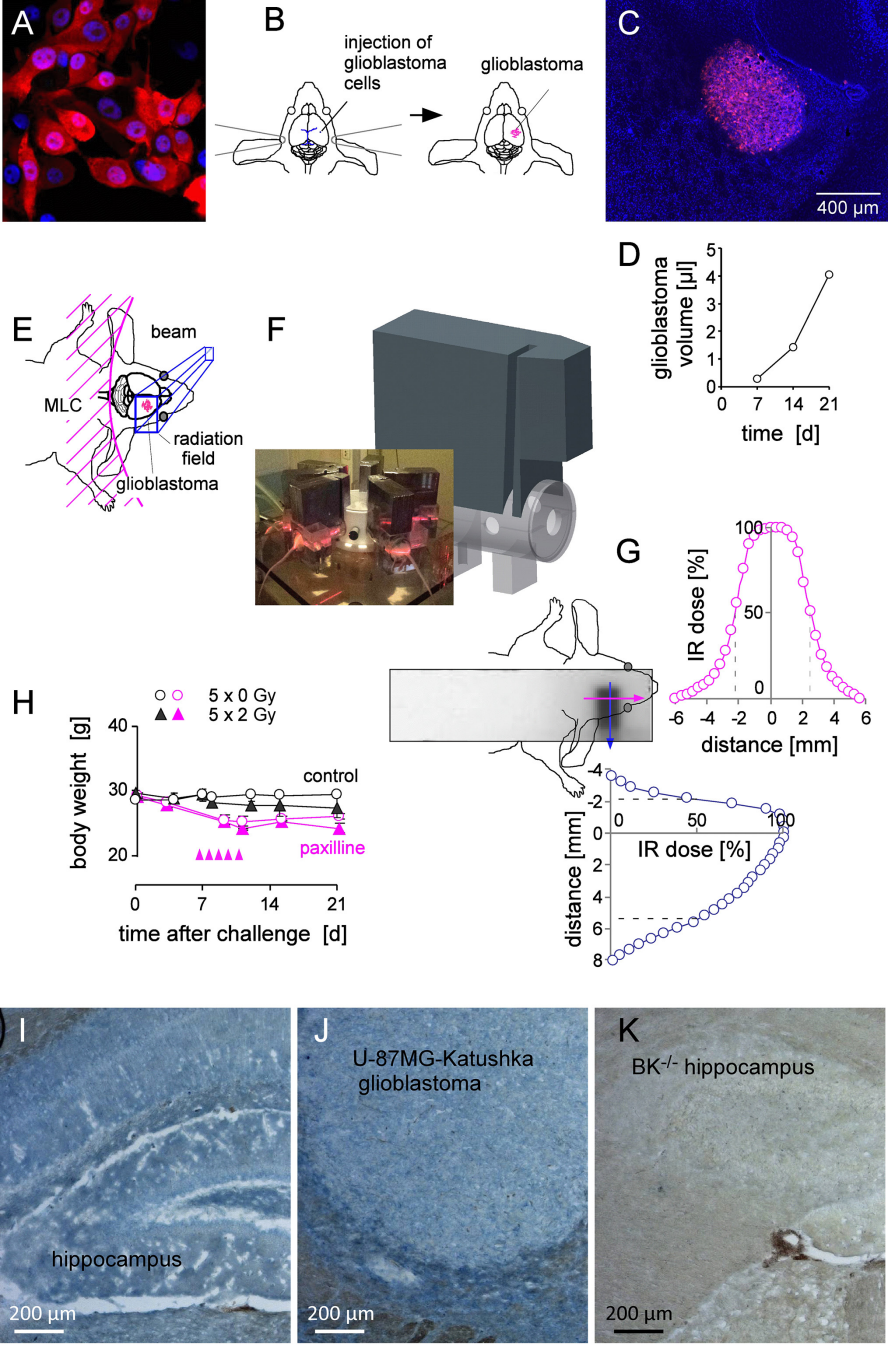
Fractionated IR of human glioblastoma xenografted orthotopically in NSG mice (**A**) Fluorescence micrograph of human U-87MG-Katushka glioblastoma cells grown *in vitro* (red and blue fluorescence indicate the Katushka protein and the DNA-specific DAPI fluorochrome, respectively). (**B**) Scheme illustrating the stereotactic transplantation of U-87MG-Katushka cells in mouse right striatum. The blue lines (left drawing) indicate bregma (top) and lambda (bottom). (**C**) Fluorescence micrograph of a U-87MG-Katushka glioblastoma in mouse brain 7 d after tumor cell challenge into the right striatum (DAPI-stained cryosection). (**D**) Time-dependent intracranial growth of U-87MG-Katushka glioblastoma. (**E**) Cartoon illustrating the radiation field. On-body lead shielding has been left out for better clarity (MLC: multileaf collimator). (**F**) Drawing of the mouse holder with mounted on-body leaf shielding. The photography on the lower left shows 6 mice during radiotherapy. (**G**) Dosimetry film and densitometrically analyzed dose distribution in y- (blue) and x-axis (pink) across the radiation field and adjacent shielded brain area. The site of dose deposition is indicated by the superimposed drawing of the mouse head. The dashed lines in the dose distribution plots indicate the 50% isodose. (**H**) Mean (± SE, *n* = 3) body weight of control (open circles) and fractionated irradiated NSG mice (closed triangles) during the first 3 weeks after intracranial challenge with U-87MG-Katushka cells. Control (black symbols) and mice receiving paxilline (pink symbols) are shown. IR fractions (2 Gy each) are indicated by the pink arrow heads. (**I, J**) BK protein expression (blue) in hippocampus and *xeno*grafted U-87MG-Katushka tumor of NSG mice. (**K**) Hippocampus of BK^+/–^ mice served as negative control.

For generation of orthotopic glioblastoma, U-87MG-Katushka (Figure 8A) cells were stereotactically inoculated into the right striatum of NSG mice (Figure 8B). Inoculation resulted in the formation of solid and most widely encapsulated glioblastoma (Figure 8C) that grew exponentially during the first 3 weeks after tumor challenge (Figure 8D). As illustrated in Figure 8E, 8F, the glioblastoma-bearing right hemisphere of isofluran-anaesthesized mice was fractionated irradiated (6 MV photons) on days 7–11 with daily fractions of 2 Gy by the use of mouse holders mounted in the radiation beam of a linear accelerator. The mouse torso and head outside the radiation field were shielded by multileaf collimator and/or an 8 cm thick on-body lead block. The film dosimetry revealed steep dose decline outside the radiation field restricting the area bounded by the 50% isodose line to about 0.8 cm × 0.5 cm *=* 0.4 cm^2^ (with about 1/3 of this field outside the animal (Figure 8F, 8G). Fractionated IR (5 × 2 Gy) was well tolerated by the mice as deduced from the only minute IR-associated decline of body weight (Figure 8H, black symbols). The mice receiving paxilline developed mild and transient ataxia and showed a body weight loss of some 10–15% as compared to the respective control groups (Figure 8H, pink symbols). After end of paxilline treatment, mice recovered completely. On day 21, mice of the 4 treatment groups were sacrificed and brains excised for histological analysis. Importantly, U-87MG-Katushka cells continued to express BK channels when grown in mouse brain as demonstrated by immunohistochemistry (Figure 8I–8K).

**Figure 9:**
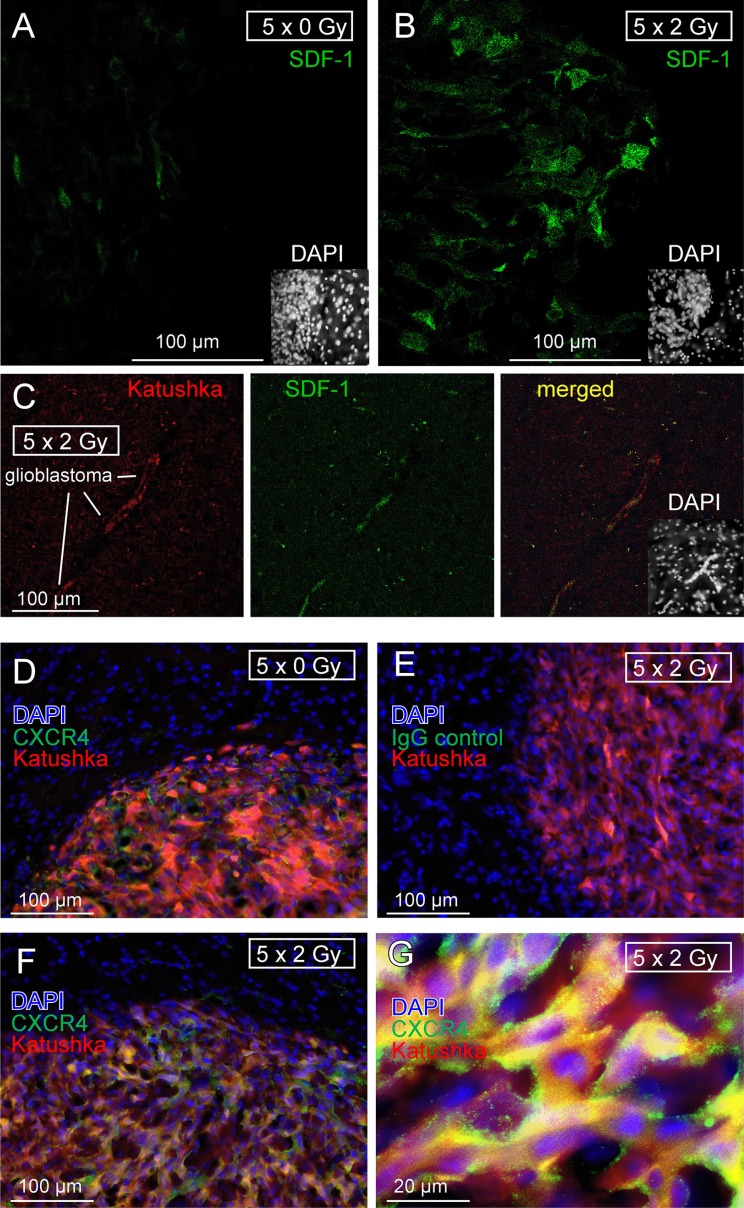
Fractionated IR stimulates *in vivo* SDF-1 protein expression by glioblastoma cells (**A, B**) SDF-1 specific immunofluorescence (green) of (A) control (5 × 0 Gy) and (B) fractionated irradiated (5 × 2 Gy) U-87MG-Katushka glioblastoma and surrounding normal brain tissue. (**C**) U-87MG-Katushka cells (red) migrating through mouse brain and expressing SDF-1 protein (green). The inserts in the lower right show the corresponding DAPI staining (white) of the nuclei in lower power. The glioblastoma in (A) and (B) can be easily identified by the dense array of nuclei. (**D–G)** U-87MG-Katushka glioblastoma expresses chemokine receptor CXCR4. CXCR4-specific immunofluorescence (green) in control (5 × 0 Gy, D) and fractionated irradiated (5 × 2 Gy) tumors (F, G; Katushka: red, DAPI: blue). CXCR4 was detectable in plasma membrane and cytoplasma of the glioblastoma cells (merged yellow Katushka and CXCR4-specific fluorescence, G). The IgG isotope control did not show green fluorescence (E).

Besides BK (see Figure 8I–8J), orthotopic U-87MG-Katuska cells expressed SDF-1 protein as evident from immunofluorescence microscopy. In particular, fractionated IR (5 × 2 Gy) induced marked up-regulation of SDF-1 protein expression 9 days after end of radiotherapy (Figure 9A, 9B). Specifically, brain invading cells at the glioblastoma margin (Figure 9B) and emigrated tumor cells that infiltrate healthy brain parenchyma were SDF-1 positive (Figure 9C) while unirradiated glioblastoma showed only weak SDF-1-specific staining (Figure 9A).

In addition to SDF-1, unirradiated and fractionated irradiated orthotopic U-87MG-Katushka cells expressed CXCR4 chemokine receptor protein (Figure 9D, 9E). CXCR4 protein abundance was similar in fractionated irradiated (Figure 9F) and in unirradiated tumors and localized in the plasma membrane (green fluorescence) and the cytoplasm (merged yellow fluorescence due to colocalization with the far-red Katushka protein, Figure 9G).

**Figure 10:**
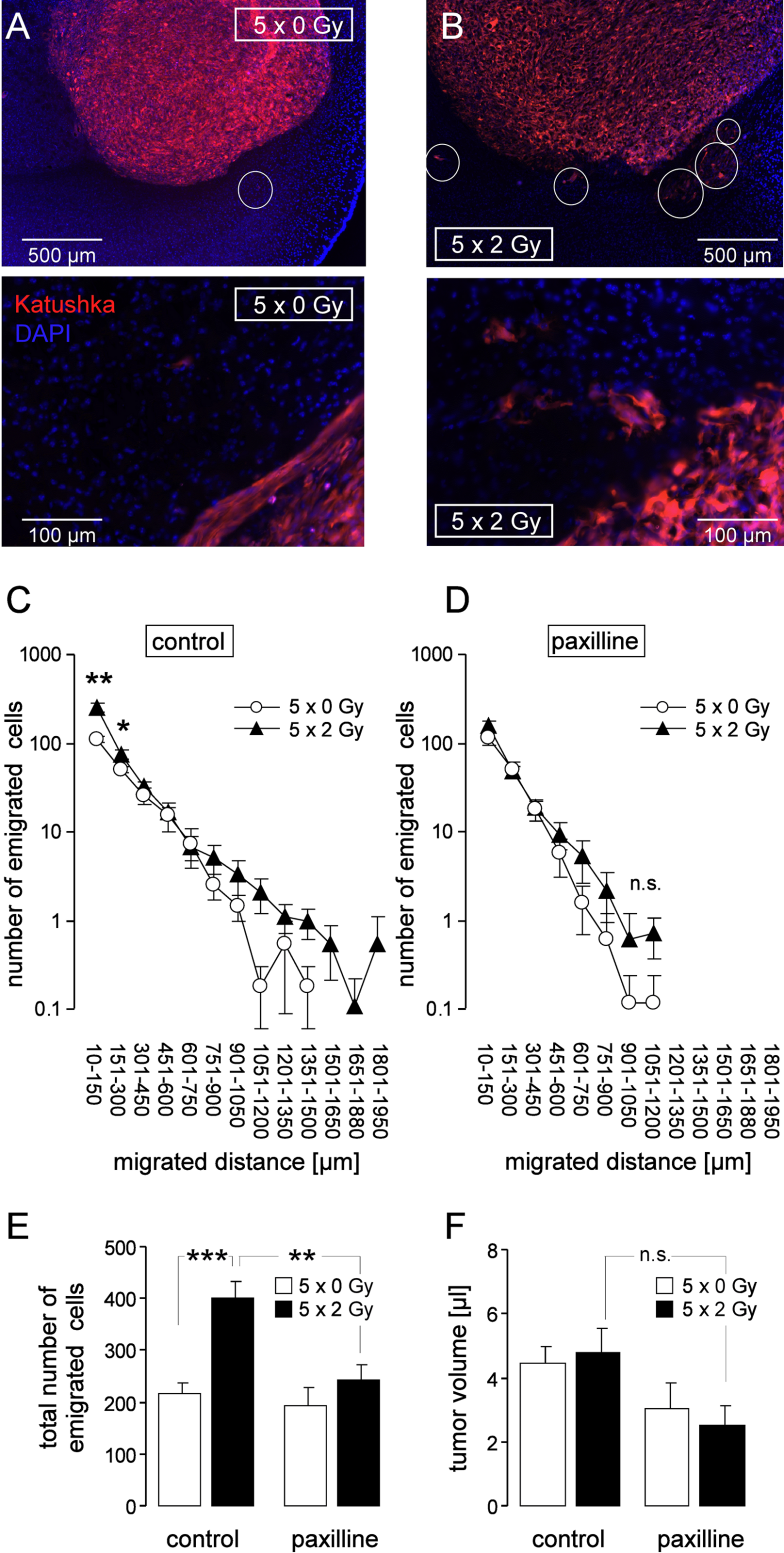
Fractionated IR stimulates migration of glioblastoma cells *in vivo* (**A, B**) Fluorescence micrographs of control (5 × 0 Gy, A) and fractionated irradiated (5 × 2 Gy, B) U-87MG-Katushka glioblastoma in low (top) and high (bottom) magnification. The nuclei are stained with DAPI (blue), some emigrating U-87MG-Katushka cells are highlighted by white circles. (**C, D**) Number of emigrated cells per tumor (mean ± SE, *n* = 8–11) as function of the migrated distance of glioblastoma cells fractionated irradiated (5 × 0 Gy, open circles or 5 × 2 Gy, closed triangles) in the absence (C) or presence (D) of concomitant BK channel targeting with paxilline. (**E, F**) Mean (± SE, *n* = 8–11) total number of emigrated glioblastoma cells (E) and mean (± SE) corresponding glioma volume (F) of fractionated irradiated glioma (5 × 0 Gy, open bars or 5 × 2 Gy, closed bars) of control mice or mice receiving concomitant paxilline chemotherapie. ***, *, **, and n.s. indicate *p* ≤ 0.05, *p* ≤ 0.01, *p* ≤ 0.001 and not significantly different, respectively, two-tailed Welch-corrected *t*-test in (C, D) and ANOVA in (E, F).

To quantify the emigration activity of untreated and irradiated tumors, the number of evaded glioblastoma cells was counted and the migration distances determined. The margin of untreated tumors was usually clearly delimited with tangentially oriented glioblastoma cells at the tumor surface (Figure 10A). Irradiated tumors (Figure 10B), in contrast, exhibited zones where glioblastoma cells invaded in the adjacent brain parenchyma giving the tumor margin a fringed appearance. Figure 10C depicts numbers and emigration distances of glioblastoma cells emigrated from unirradiated (5 × 0 Gy, open circles) and fractionated irradiated (5 × 2 Gy, closed triangles) glioblastoma. IR regimes were applied in the absence (Figure 10C) or presence (Figure 10D) of systemic paxilline administration (8 mg/kg B.W. i.p., 6 h prior to and 6 h after each IR fraction). IR significantly stimulated emigration from the tumor (Figure 10E, left) and paxilline administration prevented this IR-induced migration but did not affect basal emigration (Figure 10E, right). Importantly, neither IR nor paxilline changed the glioblastoma volume (Figure 10F).

In summary, our data on IR-induced migration of glioblastoma cells acquired *in vitro* by the use of the 2D-cultures and those obtained *in vivo* in an orthotopic glioblastoma mouse model strikingly coincided. The IR-induced induction of migration possibly occurs via IR-induced stabilization of HIF-1α. The subsequent up-regulation of the HIF-1α target gene SDF-1 was observed *in vitro* and *in vivo*, suggesting that SDF-1 can stimulate Ca^2+^ transients that lead to BK K^+^ channel activation. *In vitro* and *in vivo*, BK channel targeting prevented IR-induced migration indicating that BK is functionally involved in the stress response of irradiated glioblastoma cells.

## DISCUSSION

Whether or not IR-induced glioblastoma cell migration occurs *in vivo* is highly controversially debated and of relevance for the radiotherapy. Our study was conceptualized to get a quantitative answer to this question on cellular resolution. The new findings of our study are that in an orthotopic glioblastoma mouse model only 5 fractions of irradiation with the clinical relevant dose of 2 Gy were sufficient to increase the number of cells infiltring the brain parenchyma by factor of two. Thereby, IR-induced BK channel activity seemed to be a key event since BK blockage by paxilline abrogated IR-induced brain infiltration. Finally, our *in vivo* and *in vitro* experiments strongly suggest that IR-induced auto-/paracrine SDF-1/CXCR4 signaling contribute to BK channel activation.

Our *in vivo* glioblastoma model, U-87MG-Katushka, develops largely encapsulated gliomas in mouse brain. One might say that U-87MG cells, therefore, do not represent the majority of glioblastoma that grow highly infiltative, which certainly limits the generalization of our findings. On the other hand, the encapsulated growth of U-87MG cells only enabled us to quantitatively analyze number and migration distances of cells that emigrated out of the primary tumor lesion and infiltrated the brain. Moreover, U-87MG cells have the advantages to be tumorigenic, to quickly generate tumor mass with highly reproducible tumor volumes, to exhibit IR-induced migration *in vitro* and to express all components required for IR-induced migration as defined so far.

Another limitation of our *in vivo* study is that the applied glioblastoma treatment protocol (5 × 2 Gy) only partially reflects the trimodal therapy (surgery, 30 × 2 Gy radiotherapy and temozolomide chemotherapy) of glioblastoma patients [17]. Previously reported *in vivo* data on IR-induced migration of glioblastoma were acquired with cells or brains pre-irradiated prior to transplantation [18, 19], whole brain irradiation with a single dose of 8 Gy [10], partial brain irradiation with large irradiation fields (1 cm^2^) and single doses of 8 and 15 Gy [13], or stereotactical glioblastoma irradiation with a single dose of 50 Gy [20]. Although, each of these studies has the above mentioned limitations, these pieces of evidence combined strongly suggest that IR-induced migration is a general phenomenon and may occur during fractionated radiation therapy of glioblastoma patients.

Does IR-induced migration/infiltration contribute to the apparent high radioresistance of glioblastoma? After clinical radio(chemo)therapy, most (70–90%) volume of the recurrent glioblastoma reportedly lays within the IR target volume [2, 21, 22]. At a first glance, this suggests that the overall contribution of target volume-emigrated tumor cells on tumor recurrence - if existent - is low. On the other hand, one might argue that recurrent glioblastoma preferentially and much faster re-expand into irradiated and necrotic brain volumes than infiltrating intact brain parenchyma. Along those lines, detailed imaging analysis has suggested that significant volume of the recurrent glioblastoma lays in the outermost zone of the IR target volume, i.e., outside the initial gross tumor volume or biological target volume as defined by MRI or PET [2]. This might fit to the idea of re-settling the IR-target volume by glioblastoma cells that formerly emigrated from the gross tumor volume.

Glioblastoma migration is programmed by Ca^2+^ signaling involving CaMKII (for review see [3, 4]). Besides glioblastoma, IR-stimulated Ca^2+^ signaling has been described in leukemia [23–25] suggesting IR-induced Ca^2+^ signaling as a general phenomenon. Like IR, SDF-1 occupation of the G protein coupled chemokine receptor CXCR4 induces Ca^2+^ signaling and migration/invasion in glioblastoma [9, 26] and pancreatic cancer [27]. In particular, SDF-1 has been shown to induce Ca^2+^ release from the Ca^2+^ stores via activation of phospholipase C, and formation of inositol 1, 4, 5-trisphosphate [28]. Notably, the Ca^2+^-activated BK channels have been demonstrated to be directly linked to inositol 1, 4, 5-triphosphate receptors via lipid rafts [29]. The present study identified BK channel targeting as effective *in vivo* strategy to prevent IR-induced migration. BK channels reportedly fullfil a dual function in glioblastoma migration. They contribute, both, to cell volume changes that motorize migration (for review see [3]) and to Ca^2+^ signaling that trigger migration [14].

BK channels are expressed in neurons of the central nervous system, e.g., in hippocampus, where they can be found in pre- and postsynaptic membranes [30]. The BK channel blocker paxilline, applied in the present study, is a neurotoxin produced by the endophytic fungus *Penicillium paxilli* and causes “ryegrass staggers” in sheep which is characterized by ataxia and uncontrollable tremors [31]. Paxilline is a very specific BK channel inhibitor which works in the nanomolar range [32]. The mechanism of BK blockage is largely unknown. Paxilline has been proposed to be an allosteric inhibitor which stabilizes the closed conformation of the channel [33]. Systemic paxilline administration in our *in vivo* experiments (8 mg/kg BW, twice per day) provoked ataxia due to the blockade of BK channels in the cerebellum [31] indicating that paxilline crosses the blood-brain barrier and reached effectiv concentrations in the brain.

Paxilline at the applied dose induced besides reversible ataxia no severe side effects and was well tolerated by the mice. This might suggest that BK channel targeting might be applied in glioblastoma patients. As a matter of fact, drugs with BK channel modulating side effects are already in clinical use. Classical neuroleptics such as haloperidol or chlorpromazine inhibit BK channels with an IC_50_ in the low micromolar range [34]. Reportedly, haloperidol may accumulate in the human brain up to micromolar [35] and chlorpromazine up to several ten micromolar concentrations [36] suggesting that the therapeutic concentrations of the classical neuroleptics affect BK channel activity.

In conclusion, fractionated radiation stimulates migration of glioblastoma cells *in vivo*. This phenomenon might lead to enhanced tumor spreading during fractionated radiotherapy and might contribute to therapy failure. Radiation-induced BK K^+^ channel activation triggers and BK channel blockage suppresses IR-induced migration suggesting BK channel targeting as useful-tool to overcome IR induced migration during radiotherapy.

## MATERIALS AND METHODS

### Cell culture

Human T98G and U-87MG glioblastoma cells were from ATCC (Bethesda, Maryland, USA) and were grown in 10% fetal calf serum (FCS)-supplemented RPMI-1640 medium as described [14]. The human U-87MG glioblastoma cells were transfected with the far-red Katushka fluorescent protein expression vector pTurboFP635-N (BioCat, Heidelberg, Germany) using the transfection reagent FUGENE HD (Roche Diagnostics GmbH, Mannheim, Germany) according to the manufacturer's instruction. Stably transfected cells were grown in 10% FCS-supplemented RPMI-1640 selection medium containing G418 (750 μg/ml). Exponential growing T98G and U-87MG-Katushka cells were irradiated with 6 MV photons (IR, single dose of 0, 2, 4 and 6 Gy) or five daily fractions of 0 or 2 Gy (fractionated IR) by using a linear accelerator (LINAC SL25 Philips) at a dose rate of 4 Gy/min at room temperature. Following IR, cells were post-incubated in RPMI-1640/10% FCS medium for 2–4.5 h (immunoblot, patch-clamp, fura-2 Ca^2+^-imaging, transfilter migration, immunofluorescence microscopy), 24 h (RT-PCR, transfilter migration), or 2–3 weeks (colony formation assay). In some experiments, cells were pre-incubated (0.5 h) and post-incubated after IR with the BK channel inhibitor paxilline (5 μM, Sigma-Aldrich, Taufkirchen, Germany) or the CXCR4 chemokine receptor antagonist AMD3100 (1 μM, Sigma-Aldrich) or vehicle alone (0.1% DMSO). shRNA-transfected T98G cells were grown in RPMI-1640/10% FCS selection medium containing puromycin (2 μg/ml).

### Patch-clamp recording

Whole-cell and on-cell currents were evoked by 41 (whole-cell) or 33 (on-cell) voltage square pulses (700 ms each) from −66 mV (whole-cell) or 4 mV holding potential (on-cell) to voltages between −116 (whole-cell) or −56 mV (on-cell) and 84 (whole-cell) or +104 mV (on-cell) delivered in 5 mV increments. The liquid junction potentials between the pipette and the bath solutions were estimated as described [16], and data were corrected for the estimated liquid junction potentials. Cells were superfused at 37°C temperature with NaCl solution (in mM: 125 NaCl, 32 N-2-hydroxyethylpiperazine-N-2-ethanesulfonic acid (HEPES), 5 KCl, 5 d-glucose, 1 MgCl_2_, 1 CaCl_2_, titrated with NaOH to pH 7.4). In the whole-cell experiments shown in Figure 1, a K-d-gluconate pipette solution was used containing (in mM): 140 K-d-gluconate, 5 HEPES, 5 MgCl_2_, 1 K_2_-EGTA, 1 K_2_-ATP, titrated with KOH to pH 7.4. Paxilline (5 μM) was added to the bath solution.

For the on-cell experiments (Figures 2–4, 6–7) the pipette solution contained (in mM) 0 or 0.005 paxilline in 0.1% DMSO, 130 KCl, 32 HEPES, 5 d-glucose, 1 MgCl_2_, 1 CaCl_2_, titrated with KOH to pH 7.4. We used a high K^+^-containing pipette solution in order to have a direct quantitative measure for IK channel activity by analyzing the inward currents at highly negative voltages (since BK currents are negligible at these negative voltages). IK channel activity is also induced by radiation [16]. In some experiments, the chemokine stromal cell-derived factor-1 (SDF-1, CXCL12, 50 nM, Immuno Tools, Friesoythe, Germany) and paxilline (5 μM) was added to the bath solution. Whole-cell and macroscopic on-cell currents were analyzed by averaging the currents between 100 and 700 ms of each square pulse. Applied voltages refer to the cytoplasmic face of the membrane with respect to the extracellular space. In the current tracings (whole-cell and macroscopic on-cell currents), the individual current sweeps recorded at the different clamp-voltages are superimposed. Outward currents, defined as flow of positive charge (here: K^+^) from the cytoplasmic to the extracellular membrane face, are positive currents and depicted as upward deflections of the original current tracings.

### Colony formation assay

To test for clonogenic survival, U-87MG-Katushka and T98G cells were preincubated (0.5 h), irradiated (0, 2, 4 or 6 Gy) and post-incubated (24 h) in RPMI-1640/10% FCS medium additionally containing paxilline (0 or 5 μM in 0.1% DMSO). 24 h after IR, cells were detached, 300 and 600 cells were re-seeded in inhibitor-free medium on 3 cm wells and grown for further 2–3 weeks. The plating efficiency was defined by dividing the number of colonies by the number of plated cells. Plating efficiencies of control and paxilline-treated cells were 0.23 ± 0.001 and 0.22 ± 0.001 for T98G (*n* = 36) and 0.10 ± 0.003 and 0.06 ± 0.006 (*n* = 12) for U-87MG-Katushka cells, respectively. Survival fractions as calculated by dividing the plating efficiency of the irradiated cells by those of the unirradiated controls were fitted by the use of the linear quadratic equation.

### Transfilter migration

The lower and upper chamber of a CIM-Plate 16 (Roche, Mannheim, Germany) were filled with 160 μl (lower chamber) and 100 μl (upper chamber) of RPMI-1640 medium containing 5% and 1% FCS, respectively, equilibrated at 37°C and 5% CO_2_ for 30–60 min. The upper and lower chamber additionally contained SDF-1 (0 or 50 nM) and paxilline (0 or 5 μM in 0.1% DMSO). After CO_2_ equilibration and resetting the impedance to zero, 100 μl of cell suspension containing 40.000 of unirradiated (SDF-1 experiments) or irradiated cells (0 or 2 Gy, 1–2 h after IR) in RPMI-1640/1% FCS were added to the upper chamber. After sedimentation and adherence of the cells (2–3 h after IR), migration was analyzed in real-time by measuring the impedance increase between electrodes which cover the lower surface of the filter membrane and the reference electrode in the lower chamber. Upon trans-filter migration, cells adhere to the filter electrode surface and increase the impedance. To compare between individual experiments the impedances were normalized to the 0.5 h values of the respective controls.

### Quantitative RT-PCR

Messenger RNAs of fractionated irradiated (5 × 0 Gy or 5 × 2 Gy) U-87MG-Katushka and stably transfected T98G cells (see below) were isolated (Qiagen RNA extraction kit, Hilden, Germany) 24 h after the last IR fraction and reversely transcribed in cDNA (Transcriptor First Strand cDNA Synthesis Kit, Roche, Mannheim, Germany). BK K^+^ channel-, CXCR4 chemokine receptor-, SDF-1 (CXCL12)-, matrix metalloproteinases MMP-2- and MMP-9-, and housekeeper β-actin (ACTB)-, pyruvate dehydrogenase beta (PDHB)-, and glyceraldehyde-3-phosphate dehydrogenase (GAPDH)-specific fragments were amplified by the use of SYBR Green-based quantitative real-time PCR (QT00024157, QT00223188, QT00087591, QT00088396, QT00040040, QT01192646, QT00095431, and QT00031227 QuantiTect Primer Assay and QuantiFast SYBR^®^ Green PCR Kit, Qiagen) in a Roche LightCycler Instrument. Abundances of the individual mRNAs were normalized to the geometrical mean of the three housekeeper mRNAs.

### Western blotting

Whole protein lysates were prepared from semiconfluent irradiated (0 or 2 Gy, 2 h after IR) U-87MG-Katushka and stably transfected T98G cells (see below) using a buffer containing (in mM) 50 HEPES pH 7.5, 150 NaCl, 1 EDTA, 10 sodium pyrophosphate, 10 NaF, 2 Na_3_VO_4_, 1 phenylmethylsulfonylfluorid (PMSF) additionally containing 1% Triton X-100, 5 μg/ml aprotinin, 5 μg/ml leupeptin, and 3 μg/ml pepstatin (all Sigma-Aldrich), and separated by SDS-PAGE under reducing conditions. Segregated proteins were electro-transferred onto PVDF membranes (Roth, Karlsruhe, Germany). Blots were blocked in tris(hydroxymethyl) aminomethane-buffered saline (TBS) buffer containing 0.05% Tween 20 and 5% non-fat dry milk for 1 h at room temperature. The membranes were incubated overnight at 4°C with the following primary antibodies in TBS -Tween/5% milk against human CXCR4 (rabbit polyclonal antibody, #ab2074, 1:500 dilution, Abcam, Cambridge, UK), human HIF-1α (rabbit monoclonal, #61275, 1:1000 dilution, Active Motif, La Hulpe, Belgium) and human BK (rabbit polyclonal, #APC-107, 1:500 dilution, Alamone Labs, Jerusalem, Israel). Equal gel loading was verified by an antibody against β-actin (mouse anti-β-actin antibody, clone AC-74, Sigma #A2228 1:30,000). Antibody binding was detected with a horseradish peroxidase-linked goat anti-rabbit or horse anti-mouse IgG antibody (# 7074 and #7076, respectively; 1:2000 dilution in TBS-Tween/5% milk, Cell Signaling, Merck-Millipore, Darmstadt, Germany) incubated for 1 h at room temperature and enhanced chemoluminescence (ECL Western blotting analysis system, GE Healthcare/Amersham-Biosciences, Freiburg, Germany).

### Immunofluorescence microscopy of cultured cells

U-87MG-Katushka and T98G cells (0 or 2 Gy, 2 h after IR) were grown on object slides and irradiated with 0 or 2 Gy. Two hours after IR, cells were fixed for 15 min at room temperature with phosphate buffered saline (PBS)-containing 4% formaldehyde, 3 times rinsed with PBS for 5 min and blocked for 1 h at 21°C with PBS additionally containing 1% bovine serum albumin (BSA), 5% goat serum and 0.3% Triton X-100. Cells were then incubated with polyclonal rabbit anti-SDF-1 antibody (NBP1–19778, Novus Biologicals, R & D Systems Europe, Abingdon, UK) or rabbit IgG isotype control antibody (#12–370, Merck-Millipore, both 1 mg/ml) diluted (both 1:1000) in PBS containing 1% BSA and 0.3% Triton X-100. Thereafter, cells were rinsed 3 times for 5 min with PBS, incubated for 2 h at room temperature in the dark with goat FITC-conjugated anti-rabbit IgG antibody (1:1000, NB730-F, Novus Biologicals) diluted in PBS/1% BSA/0.3% Triton X-100, rinsed 3 times for 5 min with PBS, and coverslipped with 4′,6-diamidino-2-phenylindole (DAPI) Vectashield Antifade Mounting Medium (Vector Laboratories, Loerrach, Germany).

### Fura-2 Ca^2+^ imaging

Fluorescence measurements were performed using an inverted phase-contrast microscope (Axiovert 100; Zeiss, Oberkochen, Germany). Fluorescence was evoked by a filter wheel (Visitron Systems, Puchheim, Germany)-mediated alternative excitation at 340/26 or 387/11 nm (AHF, Analysentechnik, Tübingen, Germany). Excitation and emission light was deflected by a dichroic mirror (409/LP nm beam splitter, AHF) into the objective (Fluar x40/1.30 oil; Zeiss) and transmitted to the camera (Visitron Systems), respectively. Emitted fluorescence intensity was recorded at 587/35 nm (AHF). Excitation was controlled and data acquired by Metafluor computer software (Universal Imaging, Downingtown, PA, USA). The 340/380-nm fluorescence ratio was used as a measure of cytosolic free Ca^2+^ concentration (_free_ [Ca^2+^]_i_). U-87MG-Katushka and T98G cells were incubated with fura-2/AM (2 μM for 30 min at 37°C; Molecular Probes, Goettingen, Germany) in RPMI-1640/10% FCS medium. _free_ [Ca^2+^]_i_ was determined at 37°C during superfusion with NaCl solution (in mM: 125 NaCl, 32 HEPES, 5 KCl, 5 d-glucose, 1 MgCl_2_, 2 CaCl_2_, titrated with NaOH to pH 7.4) before and during stimulation with SDF-1 (50 nM) or conditioned NaCl solution harvested from irradiated cells. For conditioning, 250.000 cells were grown for 24 h in RPMI 1640/10% FCS. After further 24 h of serum depletion, cells were washed once with NaCl solution, overlayed with 1 ml of NaCl solution, irradiated (0 or 2 Gy) and further incubated for 2 h before harvesting the NaCl solution.

### Orthotopic mouse model of human glioblastoma

Animal experiments were carried out according to the German animal protection law and approved by the local authorities. Fluorescent U-87MG-Katushka cells (Figure 8A) were inoculated stereotactically into the right striatum of 12 week old immunocompromised male and female NOD/SCID/IL2Rγ^null^ (NSG) mice. The skullcap was trepanated 2.6 mm laterally and 0.5 mm caudally of the bregma (as indicated in the drawing of Figure 8B) by the use of a dental driller and 30.000 U-87MG-Katushka cells (in 10 μl of FCS-free EMEM medium) were injected in 3 mm depth from the dura surface into the right striatum. Starting at day 7, Isofluran-anaesthesized mice were immobilized under a 6 MV linear accelerator (LINAC SL25 Philips) and the right hemispheres were irradiated with daily fractions of 0 or 2 Gy 6 MV photons using mouse holders and shieldings as described in Figure 8E, 8F. On the days of radiation, paxilline (0 or 8 mg/kg BW i.p. in 70 μl 90% DMSO) was administered 6 h prior to and 6 h after each radiation fraction to some of the mice. In particular, 6 mice of the 5 × 0 Gy and 5 × 2 Gy control groups received vehicle alone while 14 mice were not i.p. injected. The data between the vehicle-receiving and non-receiving control mice did not differ in the 5 × 0 Gy or 5 × 2 Gy group and were pooled in each group. For dosimetry, Gafchromic 3 films (Ashland Inc., Covington, KY) placed in a mouse phantom (in 5 mm depth form the phantom surface) were exposed. By comparison with unshielded calibration films, dose distribution was defined by the film blackening inside and outside the target volume. For the dosimetry film shown in Figure 8G, background blackening (as defined by unexposed films) was subtracted.

### Immunofluorescence microscopy and immunohistochemistry of brain sections

Three weeks (in pilot experiments one or two weeks) after tumor challenge, mice were sacrificed and brains were fixed (2% paraformaldehyd in phosphate buffered solution, PBS for 24 h), cryo-protected (30% sucrose in PBS for 24 h), frozen at −80°C in Richard-Allan Scientific^™^ Neg-50^™^ Frozen Section Medium (Thermo Scientific, Germany), and cryosectioned (20 μm). For glioblastoma cell migration, cryosections were directly coverslipped in Vectashield Antifade Mounting Medium with DAPI and Katushka and DAPI fluorescence was evaluated by conventional fluorescence microscopy. For each tumor, all emigrated cells were summarized to generate one data point. The total number of emigrated cells per tumor was then compared between all four groups (5 × 0 Gy- and 5 × 2 Gy-irradiated glioblastomas in absence and presence of systemical application of BK channel inhibitor paxilline).

For SDF-1 protein immunostaining, sections were post-fixed 15 min (4% paraformaldehyde in PBS) and processed identically to the protocol described above for the cultured cells. After mounting, SDF-1-specific FITC was analyzed by confocal fluorescence microscopy. Antibody specificity was confirmed by the isotype which didn't produce any considerable fluorescence staining (data not shown). For CXCR-4 staining, the brain sections were fixed and permeabilized with 100% icecold methanol for 10 min instead of PFA fixation. As primary antibody, an anti-CXCR4 antibody (rabbit polyclonal antibody, Abcam #ab2074) was used in a 1:100 dilution.

For BK channel staining (Figure 8I–8K), sections were washed three times for 5 min with PBS, fixed and permeabilized for 10 min with 100% icecold methanol, again washed with PBS for 5 min and blocked for one hour in blocking solution (PBS containing 1% BSA, 0,2% Glycin, 0,2% Lysin, 5% goat serum and 0,3% Triton X-100). Sections were incubated overnight (4°C) with rabbit anti BKa_(674–1115)_ antibody [30], 1:500 in blocking solution and after washing three times for 5 min with PBS incubated for 1 h with the biotinylated secondary antibody, 1:200 (anti rabbit IgG, Vector Laboratories) in blocking solution. The staining was visualized with the alkaline phosphatase method and the sections were covered with Aquatex (Merck-Millipore). For positive and negative control hippocampus sections from a NSG mouse (Figure 8I) and a BK^−/–^ mouse [30] (Figure 8K) were used, respectively.

### BK knockdown

BK channels were down-regulated in T98G cells by transduction with a pool of five BK-specific MISSION^®^ shRNA Lentiviral Transduction Particles and as a control with MISSION^®^ pLKO.1-puro Empty Vector Control Transduction Particles (SHCLNV-NM_002247 and SHC001V, Sigma-Aldrich). Cells were transduced according to the provided experimental protocol positively transduced clones were selected by the use of 2 μg/ml puromycin in the culture medium. Down-regulation of BK was controlled by quantitative RT-PCR and immunoblotting (Figure 3G, insert).

### SDF-1 ELISA

T98G cells (250.000) were seeded in 75 cm^2^ cell culture flasks in RPMI-1640 medium containing 10% FCS and grown over night. Cells were washed with PBS and serum depleted for 24 h. Thereafter, cells were washed with PBS and overlaid with NaCl solution (see patch-clamp section) containing protease inhibitors (Roche, cOmplete Mini, EDTA-free, #04693159001). After 30 min incubation, cells were irradiated with 0 or 2 Gy. After further 2 h the medium was harvested and the SDF-1 concentration determined using an ELISA assay kit (R&D Systems, Human CXCL12/SDF-1 DuoSet ELISA, #DY350).

## SUPPLEMENTARY MATERIALS FIGURE


