# Supplementary material for: BK K+ channel blockade inhibits radiation-induced migration/brain infiltration of glioblastoma cells
Source: Oncotarget. 2016 Feb 16;7(12):14259–78. doi: 10.18632/oncotarget.7423 (PMC4924713; doi:10.18632/oncotarget.7423)
Supplement: Supplementary file 1 [file oncotarget-07-14259-s001.pdf]

BK K+ channel blockade inhibits radiation-induced migration/ brain infiltration of glioblastoma cells

Supplementary Materials

To characterize the far-red fluorescence protein Katushka-expressing U-87MG cells, growth kinetics (Supplementary Figure S1A) and chemosensitivity (Supplementary Figure S1B–S1C) were compared between the U-87MG-Katushka cells and the parental U-87MG glioblastoma cell line showing that both cell types behave very similar. Moreover, the chemosensitivity of U-87MG-Katushka cells to the BK K^+ channel inhibitor paxilline was compared to those against vinblastine indicating only low paxilline sensitivity.

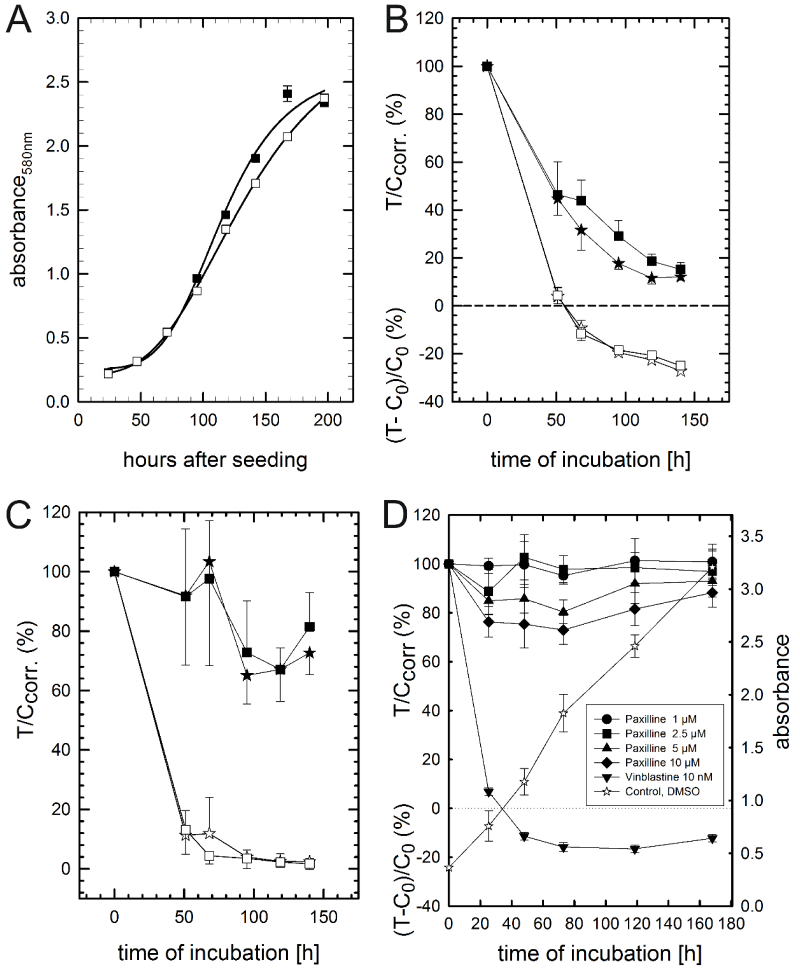

Supplementary Figure S1: U-87MG-Katushka glioblastoma cells and the parental U-87MG cell line do not differ in growth kinetics and drug sensitivity. (**A**) *In vitro* growth kinetics of parental U-87MG (open squares) and U-87MG-Katushka cells (filled squares). (**B-C**) Chemosensitivity of U-87MG parental (stars) and U-87MG-Katushka cells (squares) to (B) 50 nM (filled symbols) and 500 nM (open symbols) of mitoxantrone and (C) 25 nM (filled symbols) and 500 nM (open symbols) of topotecan. (**D**) Chemosensitivity of U-87MG-Katushka cells to paxilline and vinblastine. Cytostatic and cytotoxic drug effects are expressed as net proliferation of the treated cells referred to the vehicle control (set to 100%) and given as corrected T/C values (T/C_{corr}) where T is the mean absorbance of the treated cells and C the mean absorbance of the controls. Cytocidal effects are given as $(T-C_0)/C_0$ where T is again the mean absorbance of the treated cells and C_0 the mean absorbance of the cells at the time t_0 when the drug was added. The time-dependent increase in absorbance is shown for the control situation (open stars in D). Experimental protocols of the proliferation and cytotoxicity assay refer to Bernhardt G, Reile H, Birnböck H, Spruss T, Schönenberger H Standardized kinetic microassay to quantify differential chemosensitivity on the basis of proliferative activity. J Cancer Res Clin Oncol 1992; 118:35–41.
